# Hypertrophic neuropathy: a possible cause of pain in children with Noonan syndrome and related disorders

**DOI:** 10.1007/s00431-023-05045-6

**Published:** 2023-06-05

**Authors:** Fieke Draaisma, Corrie E. Erasmus, Hilde M. H. Braakman, Melanie C. J. Burgers, Erika K. S. M. Leenders, Tuula Rinne, Nens van Alfen, Jos M. T. Draaisma

**Affiliations:** 1 Department of Pediatrics, Radboud Institute for Health Sciences, Radboud University Medical Center, Amalia Children’s Hospital, Nijmegen, The Netherlands; 2 Department of Pediatric Neurology, Donders Institute for Brain, Cognition and Behavior, Radboud University Medical Center, Amalia Children’s Hospital, Nijmegen, The Netherlands; 3 Department of Human Genetics, Donders Institute for Brain, Cognition and Behavior, Radboud University Medical Center, Nijmegen, The Netherlands; 4 Department of Neurology, Donders Institute for Brain, Cognition and Behavior, Radboud University Medical Center, Nijmegen, The Netherlands

**Keywords:** Noonan syndrome, RASopathy, Pain, Nerve ultrasound, Hypertrophic neuropathy

## Abstract

This study is aimed at describing the findings of high-resolution nerve ultrasound in children with Noonan syndrome (NS) and related disorders experiencing pain in their legs. This retrospective cohort study was conducted in the NS expert center of the Radboud University Medical Center in the Netherlands. Patients were eligible if they were younger than 18 years, clinically and genetically diagnosed with NS or a NS related disorder, and experienced pain in their legs. Anamneses and physical examination were performed in all children. In addition, high-resolution nerve ultrasound was used to assess nerve hypertrophy and, if needed, complemented spinal magnetic resonance imaging was performed. Over a period of 6 months, four children, three with NS and one child with NS with multiple lentigines, who experienced pain of their legs were eligible for inclusion. Muscle weakness was found in two of them. High-resolution nerve ultrasound showed (localized) hypertrophic neuropathy in all patients. One child underwent additional spinal magnetic resonance imaging, which showed profound thickening of the nerve roots and plexus.

*Conclusion*: In the four children included with a NS and related disorders, pain was concomitant with nerve hypertrophy, which suggests an association between these two findings. The use of high-resolution nerve ultrasound and spinal magnetic resonance imaging might result in better understanding of the nature of this pain and the possible association to nerve hypertrophy in patients with NS and related disorders.

**Table:** 

**What is Known:** *• Children with Noonan syndrome and related disorders may report pain in their legs, which is often interpreted as growing pain.* *• Some adults with Noonan syndrome and related disorders have hypertrophic neuropathy as a possible cause of neuropathic pain.*	
**What is New:** *• This is the first study using high-resolution nerve ultrasound in children with Noonan syndrome and related disorders experiencing pain in their legs.* *• Hypertrophic neuropathy was diagnosed as possible cause of pain in four children with Noonan syndrome and related disorders.*

**What is Known:**

*• Children with Noonan syndrome and related disorders may report pain in their legs, which is often interpreted as growing pain.*

*• Some adults with Noonan syndrome and related disorders have hypertrophic neuropathy as a possible cause of neuropathic pain.*

**What is New:**

*• This is the first study using high-resolution nerve ultrasound in children with Noonan syndrome and related disorders experiencing pain in their legs.*

*• Hypertrophic neuropathy was diagnosed as possible cause of pain in four children with Noonan syndrome and related disorders.*

## Introduction

Noonan syndrome (NS) and related disorders are a group of genetically and phenotypically related conditions. NS and related disorders are caused by heterozygous pathogenic germline variants in genes belonging to the RAS-MAPK pathway, leading to RAS and ERK activation [1–3]. In this group, NS is the syndrome with the highest prevalence. Other NS-related disorders are NS with multiple lentigines (NSML), NS with loose anagen hair, NS-like disorder, cardiofaciocutaneous syndrome, and Costello syndrome. Multiple genes implicated in NS have been described, but in about 50% of patients, the *PTPN11* gene is affected [1, 2]. NS and related disorders are characterized by symptoms such as variable developmental delay, congenital cardiac defects, short stature, and facial dysmorphism. However, other potential features are also involved [2]. Although pain is frequently reported in patients with NS, this is not recognized as one of the main features [2]. Moreover, pain is particularly reported in adulthood, even though the onset of chronic pain is often during childhood [4, 5]. Children describe this pain as myalgia, predominantly in their legs, which is often interpreted as growing pain [5]. The nature and pathophysiology of this pain are still unclear. The RAS-MAPK pathway has been reported to play a role in both central and peripheral pain sensations [6]. However, a recent study in adults with NS and related disorders with intractable neuropathic pain suggested hypertrophic neuropathy as a possible cause for this pain [7]. We were interested in whether this could also be found in children with NS and related disorders who experienced pain in their legs.

## Materials and methods

This retrospective cohort study was conducted between June 2022 and January 2023 in the NS expert center of the Radboud University Medical Center in the Netherlands. Patients were eligible if they were younger than 18 years, clinically and genetically diagnosed with NS or a NS related disorder, and experienced pain in their legs. Anamnesis and physical examination, including neurologic examination, were performed in all patients. Muscle strength was evaluated using the Medical Research Council (MRC) scale for muscle strength grading. A maximum score of 5 indicates normal strength. All information regarding patient characteristics and their physical exam, high-resolution nerve ultrasound imaging, and spinal magnetic resonance imaging (MRI) was extracted from our electronic health record system (Epic Systems, Verona, WI, USA). The Medical Ethics Committee at Radboud University Medical Center Nijmegen has confirmed that no ethical approval was required for this study (file number 2023–16,170). Informed consent was acquired from all parents and, when appropriate, also from patients aged 12 years or older.

## Results

Between June 2022 and January 2023, four children, out of 21 (19%) children seen in our expert center, of three families with NS or NS‐related disorder experienced pain in their legs.

Patient 1 is a 14-year-old female patient diagnosed with NS within the first year of life based on a combination of her family history and clinical features, including facial dysmorphism, feeding problems, and delayed motor development milestones. Whole exome sequencing showed a maternally inherited, heterozygous, pathogenic variant in *PTPN11* (c.172A > G (p.Asn58Asp)). At the age of 11 years, she started to report progressive paroxysmal cramping pain in the legs, predominantly in the right leg, resulting in exercise intolerance. She had difficulties climbing the stairs at school. Neurological examination of the right leg demonstrated both proximal and distal muscular weakness of the quadriceps femoris, hamstring, and gastrocnemius muscles (MRC Grade 4). High-resolution nerve ultrasound showed extensive hypertrophic neuropathy of the sciatic nerve from the sciatic foramen down to the proximal thigh.

Patient 2 is the 10-year-old, younger brother of patient 1. He was also diagnosed with NS in the first year of his life and had the familiar pathogenic variant in the *PTPN11* gene. Like his sister, he experienced feeding problems and showed a notable delay in motor development. At the age of 10 years, he started to experience exercise-related paroxysmal cramping pain in the legs, which was explained as growth pains. Paracetamol was effective in managing the pain. The neurological examination was normal. High-resolution nerve ultrasound of the lower extremities showed likewise localized hypertrophic neuropathy of the sciatic nerve.

Patient 3 is a 13-year-old female diagnosed with NS in early childhood. WES analysis at the age of 2 years revealed a heterozygous de novo pathogenic *PTPN11* variant (c.172A > G (p.Asn58Asp)). At the age of 13 years, she was first seen in our expert center, reporting long-standing pain in both the upper and, more predominantly, lower limbs. The nature of the pain was suggestive for neuropathic pain, as she described a paroxysmal burning sensation and areas sensitive to touch. The neurological examination was normal. High-resolution nerve ultrasound showed normal nerve diameters in the upper extremities, but localized hypertrophic neuropathy of the distal sciatic and tibial nerves in the lower extremities.

**Fig. 1:**
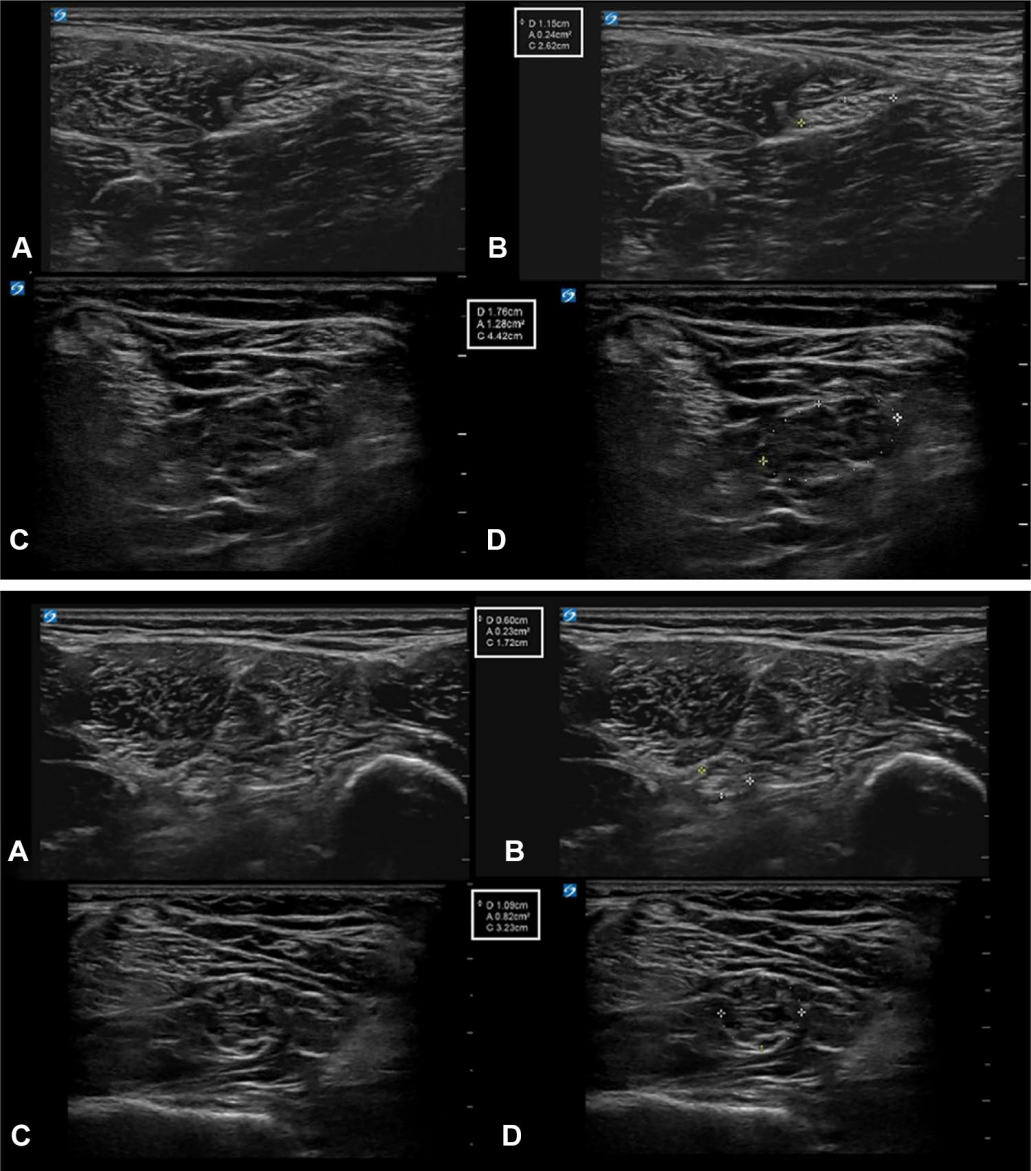
Nerve ultrasound images of patient 4 (**C** and **D**) compared to a control individual (**A** and **B**). Upper panel: the proximal sciatic nerves without (**A** and **C**) and with (**B** and **D**) cross-sectional areas. The normal nerve has a size of 24 mm^2^, which is a normal size for a 10-year-old child, while the enlarged nerve in the 10-year-old girl is 128 mm^2^. Lower panel: the distal sciatic nerves without (**A** and **C**) and with (**B **and **D**) cross-sectional areas. The normal nerve has a size of 23 mm^2^, which is a normal size for this 10-year-old child, while the enlarged nerve in a 10-year-old girl is 82 mm^2^

Patient 4 is a 10-year-old female diagnosed with NSML based on a de novo in-frame deletion-insertion in *KRAS* (c.194_195ins21 (p.Ser65delins8)). In an earlier study, this female was described to have NS, but subsequently, she developed multiple lentigines [8]. From an early age, she experienced pain in both hands and feet and developed a stepping gait. As a result, at the age of four, she could only walk inside the house but was wheelchair dependent outdoors. One year later, she was diagnosed with diffuse sensorimotor polyneuropathy with demyelinating features and, due to progressive function loss, she now fully depended on her parents for daily life activities. At the age of 10, she was seen in our expert center describing burning and tantalizing pain in both the upper and lower extremities. Neurological examination was as follows: there was atrophy of the hand musculature and calf muscles. Muscle weakness was found in both the upper and lower extremities. There was muscle weakness of the arms (MRC Grade 4); she could not spread her fingers (MRC Grade 0–1) and could only slightly extend dig 2 (MRC Grade 4). There was reduced opposing of the thumbs (MRC Grades 3–4). Moreover, there was muscle weakness of the hip and knee extensors (MRC Grade 4), the hamstrings (MRC Grades 4–5), foot lifters (MRC Grade 1), peronei (MRC Grade 0), and foot extensors (MRC Grades 3–4). In the lower extremities, gnostic and vital sensations were lowered and there were absent tendon reflexes. In the upper extremities, this was globally intact. High-resolution nerve ultrasound showed hypertrophic neuropathy of the right brachial plexus from the extraforaminal roots C5–T1 to the infraclavicular region and the right sciatic and tibial nerves (Fig. 1). No ultrasound was performed of the contralateral side. The spinal MRI showed, performed under general anesthesia, profound enlargement of the extraforaminal spinal nerve roots and both brachial and lumbar plexuses, without signs of entrapment in the neuroforamina. Since peripheral demyelinating neuropathy is uncommon among patients with NS and related disorders, further whole exome sequencing for hereditary neuropathy was performed, but no genetic abnormality was found.

## Discussion

We report three children with NS and one with NSML experiencing pain with or without muscle weakness of the limbs. High-resolution nerve ultrasound showed hypertrophic neuropathy in all four cases. This is in accordance with earlier findings in adults with NS [7]. However, only a few studies report on this subject in children. One of the adults described by de Ridder et al. with NS due to pathogenic biallelic *LZTR1* variants (c.1687G > C (p.Glu563Gln)) and c.2090G > A (p.Arg697Gln)) experienced persistent burning pain in the neck and shoulders, back, and hands and feet starting at the age of 15 years. High-resolution nerve ultrasound showed extraforaminal hypertrophic neuropathy [7]. Conboy et al. reported on a 16-year-old girl with NSML due to a familial pathogenic *PTPN11* variant (c.836A > G (p.Thr279Cys)) in which spinal MRI showed mild thickening of the caudal lumbar nerve roots [9]. Several nerve overgrowth types are described in NS and related disorders, including neurofibromas, schwannomas, and Schwann cell proliferation [7]. Pain is frequently reported in children with NS and related disorders [4, 5]. Peripheral neuropathic pain, which is characterized by burning or shooting pain, is due to damage or disease of nerves, for example, nerve entrapment [7]. We can only hypothesize on the cause of the (neuropathic) pain in our patients. As known, painful syndromes are associated with different glial activation states, including glial reaction resulting in changes of glial markers and morphology (e.g., hypertrophy) and upregulation of the MAPK pathways [6, 10]. We hypothesize that the activation of the RAS-MAPK pathway in our patients has led to hypertrophic neuropathy and pain, whereby the hypertrophic neuropathy also might be a potentiating factor for this pain. The association between germline mutations and schwannomatosis-related pain supports this hypothesis [11].

Enlarged spinal nerve roots are also features of specific pathogenic variants in neurofibromatosis 1 [12, 13]. Neurofibromatosis type 1 also gives overactivation of the RAS-MAPK pathway. MEK-inhibitors used in patients with neurofibromatosis type 1 is associated with reduced pain [14]. So it might be possible that treatment with a MEK inhibitor might also release pain in patients with NS and related disorders with hypertrophic neuropathy.

In conclusion, in children with NS and related disorders experiencing pain in their legs, hypertrophic neuropathy might be present and is probably underreported. The use of high-resolution nerve ultrasound and whole body or spinal MRI might result in better understanding of the nature of this pain and the possible association to hypertrophic neuropathy*.* The extent to which hypertrophic neuropathy appears in children without pain in their legs has yet to be established. To study this concept in clinical practice, we suggest performing high-resolution nerve ultrasound in children with and without pain. Although whole-body or spinal MRI examination may be used as a screening instrument as in neurofibromatosis type 1, this is not the preferable screening instrument in children due to the need of general anesthesia [12, 13].

## Data Availability

The data of this study are available on request from the corresponding author.
